# Athermal ω Phase and Lattice Modulation in Binary Zr-Nb Alloys

**DOI:** 10.3390/ma15062318

**Published:** 2022-03-21

**Authors:** Mitsuharu Todai, Keisuke Fukunaga, Takayoshi Nakano

**Affiliations:** 1 Department of Environmental Materials Engineering, National Institute of Technology, Niihama College, 7-1 Yagumo-cho, Niihama 792-8580, Ehime, Japan; m.todai@niihama-nct.ac.jp; 2 Division of Materials and Manufacturing Science, Graduate School of Engineering, Osaka University, 2-1 Yamada-oka, Suita 565-0871, Osaka, Japan; keisuke.fukunaga@mat.eng.osaka-u.ac.jp

**Keywords:** magnetic resonance imaging (MRI), transmission electron microscopy (TEM), phase transition, electrical resistivity, metallic biomaterials

## Abstract

To further explore the potential of Zr-based alloys as a biomaterial that will not interfere with magnetic resonance imaging (MRI), the microstructural characteristics of Zr-xat.% Nb alloys (10 ≤ x ≤ 18), particularly the athermal ω phase and lattice modulation, were investigated by conducting electrical resistivity and magnetic susceptibility measurements and transmission electron microscopy observations. The 10 Nb alloy and 12 Nb alloys had a positive temperature coefficient of electrical resistivity. The athermal ω phase existed in 10 Nb and 12 Nb alloys at room temperature. Alternatively, the 14 Nb and 18 Nb alloys had an anomalous negative temperature coefficient of the resistivity. The selected area diffraction pattern of the 14 Nb alloy revealed the co-occurrence of ω phase diffraction and diffuse satellites. These diffuse satellites were represented by ***g***_β_ + ***q*** when the zone axis was [001] or [113], but not [110]. These results imply that these diffuse satellites appeared because the transverse waves consistent with the propagation and displacement vectors were ***q*** = <ζ $\bar{\zeta }$ 0>* for the ζ~1/2 and <110> directions. It is possible that the resistivity anomaly was caused by the formation of the athermal ω phase and transverse wave. Moreover, control of the athermal ω-phase transformation and occurrence of lattice modulation led to reduced magnetic susceptibility, superior deformation properties, and a low Young’s modulus in the Zr-Nb alloys. Thus, Zr-Nb alloys are promising MRI-compatible metallic biomaterials.

## 1. Introduction

Magnetic resonance imaging (MRI) is a very useful tool in the fields of orthopedic and brain surgery, because it can yield various cross-sectional images of the human body [1]. However, the magnetic properties of metallic implants must be taken into consideration during their design, because MRI entails the application of a high magnetic field to obtain the images. Unsharp parts (artifacts) occur in the MRI data around stainless-steel and Co-Cr alloy implants, because these alloys have high magnetic susceptibility. The artifact is caused by the heat generation from and displacement of the metallic implants [2] in a high magnetic field. Thus, to reduce artifacts and obtain clear images, it is necessary to develop a metallic implant with low magnetic susceptibility [3].

Zr-based alloys have emerged as promising candidates for application as biomaterials that would result in fewer MRI artifacts [4]. This is because of their good biocompatibility [5,6], superior corrosion resistance [7], and lower magnetic susceptibility than stainless steel and Co-Cr alloys. Nomura et al. reported on the relationship between the phase constitution and magnetic susceptibility of as-cast Zr-Nb alloys at room temperature; they suggested the magnetic susceptibility of Zr-Nb alloys can be reduced by controlling the volume fraction of the athermal ω phase [8,9].

The ω phase is formed when Ti, Zr, and Hf are subjected to a high hydrostatic pressure [10,11]. This phase was also found to be metastable when β-stabilizing elements such as Nb and Ta, etc., were added to the Ti and Zr [12]. The crystal structure of this phase belongs to the space group P-3m1 [13,14], and it is known to occur as the athermal ω phase [15,16,17,18,19], isothermal ω phase [19,20], or stress-induced ω phase [12,21]. The athermal ω phase is associated with rapid cooling from the β phase region to room temperature. The isothermal ω phase is associated with isothermal aging within the approximate temperature range of 303–577 K. The precipitation of the ω phase is known to be related to the magnetic susceptibility and mechanical properties of Zr-Nb alloys. Particularly, the precipitation of the ω phase in Zr-based alloys leads to extreme brittleness and must therefore be controlled to realize high resistance to fracture and good fatigue performance [12,20]. In addition, the Young’s modulus can be decreased by suppressing the athermal ω phase. In Ti single crystals with a low *e/a* (i.e., low average number of valence electron per atom in the free-atom configuration), the Young’s modulus in the <001> direction in the unstable β phase has been found to be similar to that of human bone [22,23,24,25]. The lattice softening of *c*’ in the unstable β phase in Ti alloys has been reported to reduce the Young’s modulus in the <001> direction. In previous papers, we also reported on the occurrence of lattice modulation in Ti-Nb alloys under the combined effects of an unstable β phase and a low *e/a* [25,26]. Considering the similarity between the athermal ω phase transformation in Ti-Nb alloys and Zr-Nb alloys, we expected that, in the case of Zr-Nb alloys, an unstable β phase with lattice modulation would be associated with the lattice softening of *c*’ and a low Young’s modulus. The value of *e/a* decreases with decreasing Nb content in Zr-Nb alloys. Furthermore, the volume fraction of athermal ω phase increases with decreasing Nb content [8,9]. Thus, it is essential to find a Zr-Nb alloy with a low *e/a*, lattice modulation and no ω phase. Although the realization of Zr-Nb alloys with acceptable magnetic properties and good mechanical properties requires an understanding of the significance of the volume fraction of athermal ω phase and the details of the lattice modulation, the athermal ω phase transformation and the occurrence of lattice modulation in Zr-xNb alloys have yet to be clarified. Nomura et al. reported that the α + β + ω phase exists in Zr-6 at.% Nb alloys, and β + ω phase exists in the upper 9 at.% Nb alloys [8]. They also reported that the ω phase was not detected in the XRD patterns of the upper 12 at.% Nb alloys.

Thus, the purpose of this study was to clarify the relationship between the metastable transition and magnetic susceptibility of Zr-x at.% Nb alloys (10 ≤ x ≤ 18) by conducting electrical resistivity and magnetic susceptibility measurements and transmission electron microscopy (TEM) observations to examine their physical properties. Considering that the precipitation of the athermal ω phase affects the magnetic susceptibility, which is closely related to the electronic structure in Zr-xNb alloys, electrical resistivity measurements is appropriate for investigating athermal ω phase transformation. The Vickers hardness test was also applied, because it can sensitively detect the precipitation of the athermal ω phase.

## 2. Materials and Methods

Master ingots of the Zr-x at.% Nb (x = 10, 12, 14 and 18) alloys were prepared by an arc melting method. The ingots were melted on a water-cooled coper hearth in a high-purity Ar gas atmosphere. The chemical compositions of the ingots are listed in Table 1.

The compositions of the master ingots were analyzed by inductively coupled plasma–optical spectroscopy. The actual compositions of all examined alloys were confirmed to be nearly the same as the corresponding nominal compositions. These master ingots were remelted at least five times to prevent segregation. The ingots were subjected to a homogenization heat treatment at 1273 K for 24 h in quartz tubes, followed by quenching in ice water. The specimens to which the electrical resistivity measurements, magnetic susceptibility measurements, Vickers hardness tests, and TEM observations were to be applied were cut from the master ingots in quartz tubes prior to being subjected to a solution heat treated at 1273 K for 1 h. The specimens also were quenched in ice-cold water after solution heat treatment. According to the phase diagram for the Zr-Nb alloy system, only the β phase exists in these alloys at 1273 K. Note that, in this paper, each alloy is referred to based on its Zr content. For example, the Zr-10 at.% Nb alloy is referred to as the 10 Nb alloy.

The specimens of all examined measurements were polished with emery paper up to #2000 and then electropolished with a solution of 92 vol.% methanol and 8 vol.% perchloric acid at approximately 250 K. The electrical resistivity was measured by applying a standard four-probe method under the conditions of cooling from 300 to 4.2 K at a rate of approximately 1 K/min. The dimensions of the electrical resistivity measurement specimens were 2 mm × 10 mm × 0.2 mm. The magnetic susceptibility (χ) was measured by operating a superconducting quantum inference device magnetometer (Quantum Design; SQUID) with an external magnetic field of *μ*_0_*H* = 2 T and a constant cooling rate of approximately 1 K/min. The dimensions of magnetic susceptibility measurement specimens were 10 mm × 10 mm × 10 mm. The micro-Vickers hardness tests were performed at room temperature. The applied load was 2.94 N, and the loading time was 15 s. Specimens for TEM observations cut into a circle with a diameter of 3 mm from the ingots and then were polished to a thickness below 200 μm by emery paper. Finally, a thin foil was prepared for the TEM observations by using the twin-jet technique. TEM was performed by operating a JEM 3010 (JEOL) at 300 kV.

## 3. Results

### 3.1. Electrical Resistivity Measurements

**Figure 1:**
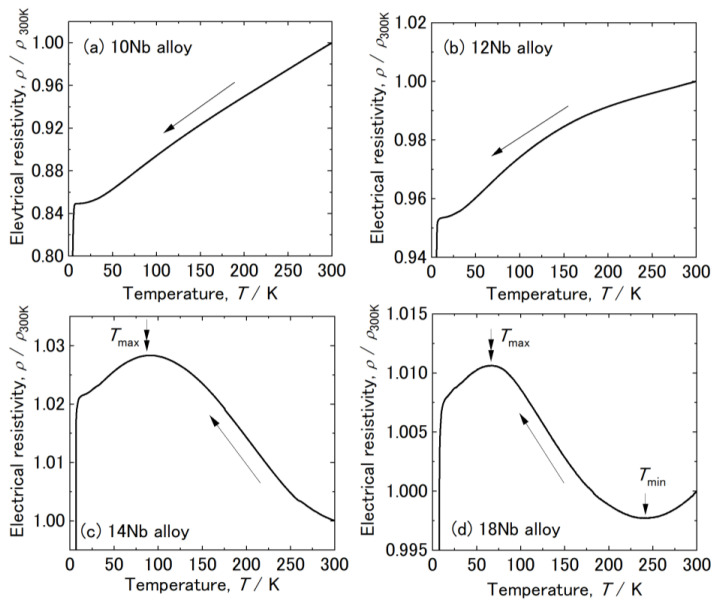
Electrical resistivity of various Zr-x at.% Nb alloys (x = 10, 12, 14, and 18) during the cooling process: (**a**–**d**) x = 10, 12, 14, and 18, respectively.

Figure 1 shows the temperature dependence of the electrical resistivity of various Zr-x at.% Nb alloys (x = 10, 12, 14, and 18).The resistivity of the 10 Nb and 12 Nb alloys monotonically decreased with decreasing temperature. The analysis of the resistivity curve for the 14 Nb alloy revealed negative temperature coefficients (NTCs) at temperatures below room temperature. This NTC has previously been confirmed for several Ti alloys [27,28,29,30]. In previous studies, an anomalous NTC in the resistivity curve was interpreted as the growth of the athermal ω phase and occurrence of lattice modulation during the cooling process [29,31]. This result indicated that lattice modulation would occur in the β phase of the 14 Nb alloy. The resistivity of the 14 Nb alloy began decreasing again at *T*_max_ = 100 K, as indicated by the double arrow in Figure 1c. The resistivity curve for the 18 Nb alloy was found to have a local minimum at *T*_min_ = 240 K, as indicated by the arrow, and an NTC below *T*_min_. The resistivity of this alloy began decreasing again at *T*_max_ = 70 K. These results revealed that, *T*_min_ and *T*_max_ decreased with increasing Nb content. Furthermore, the composition dependence and transformation behavior observed in this study were found to be similar to those previous reports for some β phase in Ti alloys [27,29,31].

**Figure 2:**
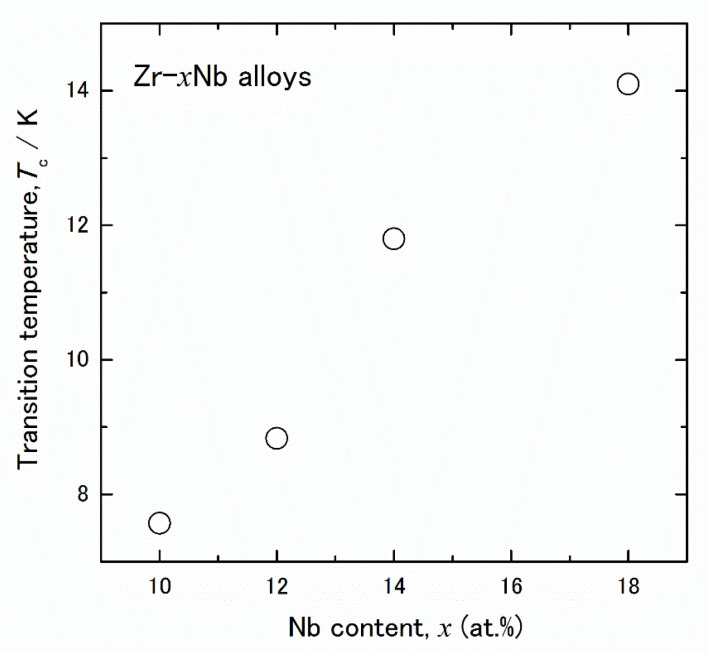
Nb content dependence of the superconductive transition temperature (*T*_c_) of Zr-x at.% Nb (10 ≤ x ≤ 18) alloys.

Additionally, a sharp decrease in the electrical resistivity was observed for all alloys at temperatures below 15 K. These drops in resistivity were induced by the superconductive transition. As shown in Figure 2, the superconductive transition temperature, *T*_c_, of these alloys increased with increasing Nb temperature. The error bar is almost the same as in the plots. Generally, the *T*_c_ of Zr-Nb alloys is higher than that of Ti-Nb alloys [26,31]; here, its value changed as the Nb content increased. These results indicate that the phase constitution gradually changed as the Nb content increased.

### 3.2. TEM Observations

**Figure 3:**
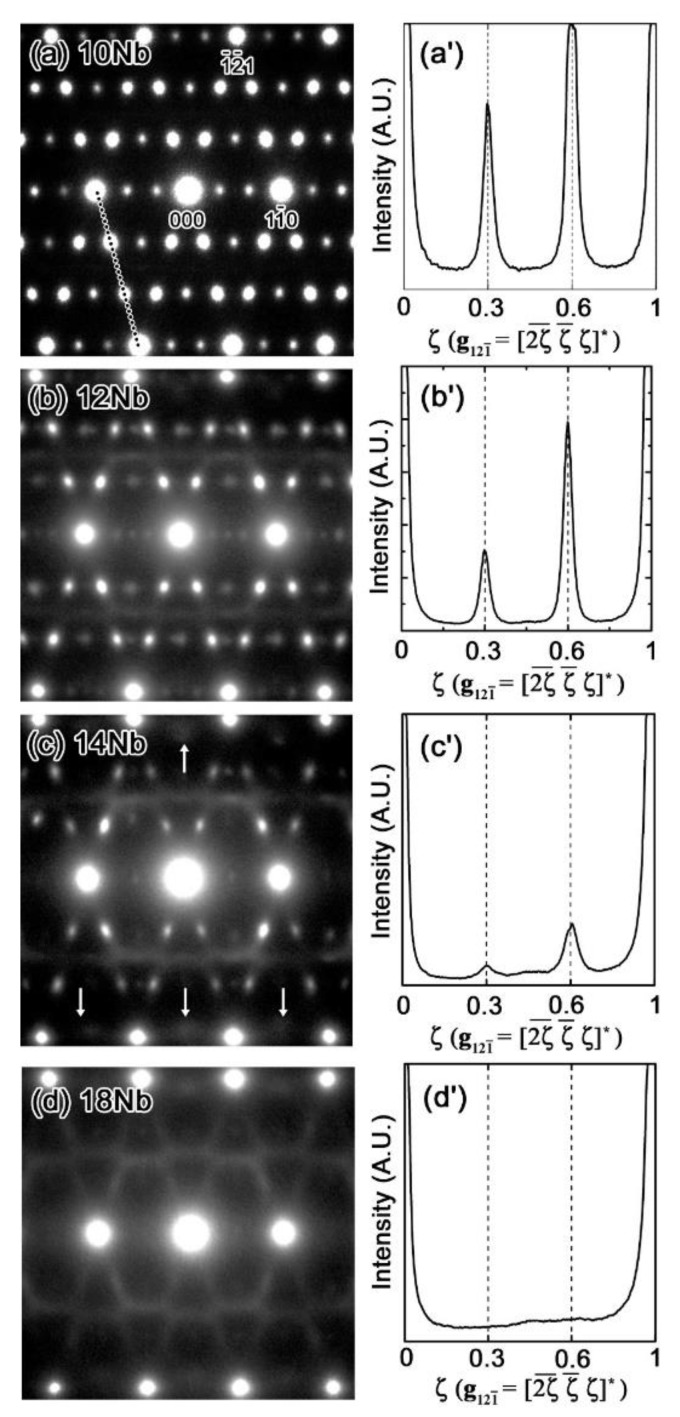
Results for Zr-x at.% Nb alloys (10 ≤ x ≤ 18), as obtained by applying the incident beam in the [113] direction at room temperature: (**a**–**d**) diffraction patterns for x = 10, 12, 14, and 18, respectively; (**a’**–**d’**) corresponding intensity profiles.

Figure 3a–d show the selected area diffraction patterns (SADPs) of the Zr-x at.% Nb (10 ≤ x ≤ 18) alloys, as obtained under the conditions of a beam direction of [113] at room temperature.

**Figure 4:**
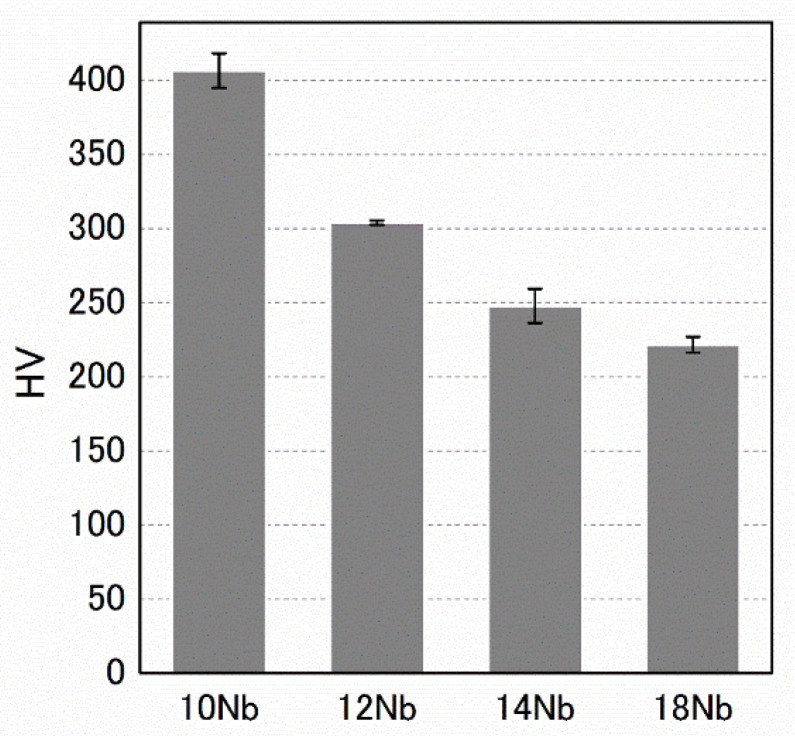
HV variation of the Zr-x at.% Nb alloys.

The diffraction pattern of the 10 Nb alloy indicated the existence of the β phase and additional spots at ***g***_β_ + 1/3<2ζ ζ ζ>* and ***g***_β_ + 2/3<2ζ ζ ζ>*. Here, ***g***_β_ is the β-phase reciprocal lattice vector, and the asterisk (*) indicates the orientation in the reciprocal space. Additional spots have been reported to be associated with the diffraction pattern of the athermal ω phase [12]; here, they appeared in the SADPs of the 10 Nb, 12 Nb and 14 Nb alloys. It should be noted that the intensity of the ω-phase spot decreased with increasing Nb content. Additionally, these diffraction patterns did not show clearly defined spots, and only diffuse streaks were observed in the case of the 18 Nb alloy. This behavior can be better understood by comparing the one dimensional intensity profiles along [$\bar{2}\bar{1}$1]*, as shown in Figure 3a′–d′. These intensity profiles show that additional spots appeared at the commensurate ***g***_β_ + 1/3<2ζ ζ ζ>* and ***g***_β_ + 2/3<2ζ ζ ζ>* positions, and that their intensity decreased with increasing Nb content. In addition, Figure 4 shows that HV decreases with increasing Nb content. These results indicate that the volume fraction of the athermal ω phase gradually decreased with increasing Nb content; moreover, these results were in good agreement with the results of the resistivity measurements.

**Figure 5:**
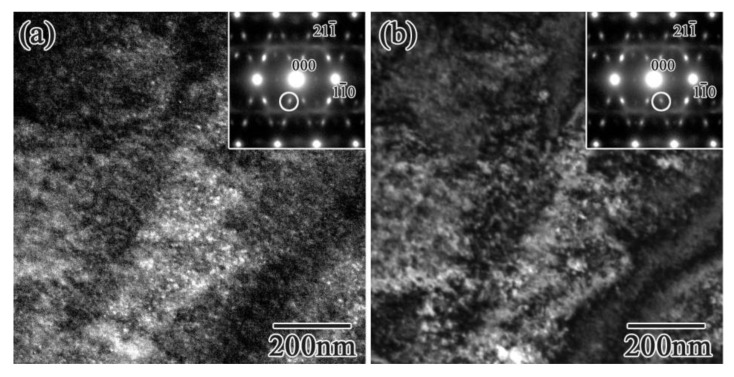
Dark-field images of Zr-14 at.% Nb alloy were observed by a circle in diffraction patterns in (**a**,**b**), respectively.

To understand the morphology of the athermal ω phase, dark-filed images of the ω phase spot in the 14 Nb alloy were obtained; the results are shown in Figure 5.

The athermal ω phase appeared as an approximately 10 nm–diameter sphere, which is in agreement with a previous reported results [32]. The density of the precipitated athermal ω phase decreased with increasing Nb concentration, although the size showed little Nb dependence. The shape of precipitated athermal ω phase in Zr-x at.% Nb alloys was found to be the same as that in Ti alloys [12] and other Zr based alloys [16]. It should also be noted that, in the 14 Nb alloy, the rod-like streaks along [110]*, as indicated by the white arrows in Figure 3c, and the weak diffuse satellite near ***g***_12$\bar{1}$_ + 1/2[ζ$\bar{\zeta }$0]* are indicated by the appearance of lattice modulation.

## 4. Discussion

To investigate the occurrence of lattice modulation in the β phase, TEM was applied to the 14 Nb alloy and observed by TEM, according to the results of Ti alloys [31,33,34].

**Figure 6:**
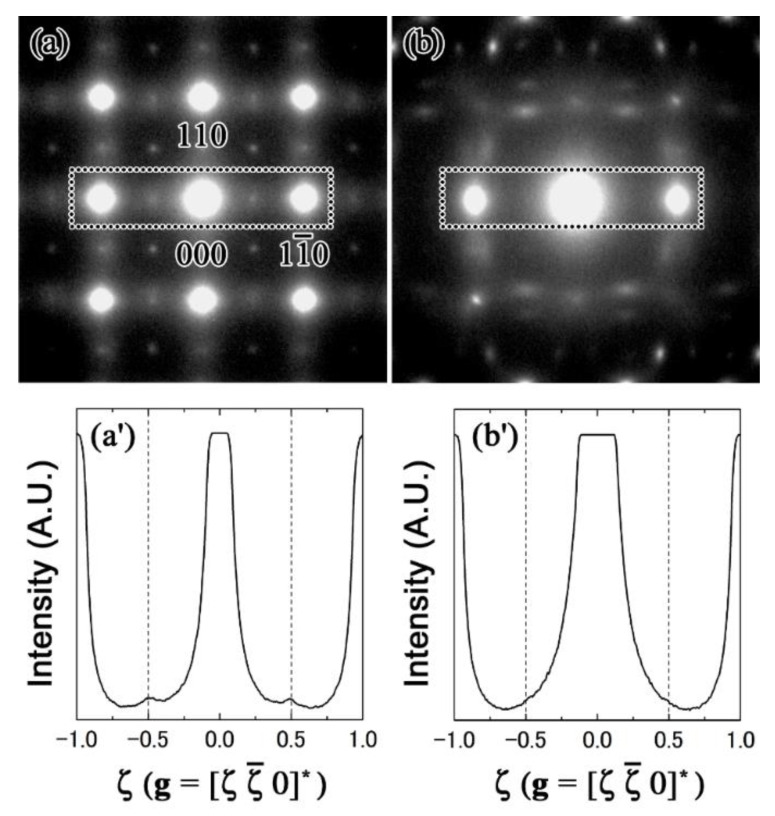
SADPs of the Zr-14 at.% Nb alloy: (**a**) SADP showing the zone axis of [001]; (**b**) SADP showing the systematic condition; (**a’**,**b’**) corresponding intensity profiles for the respective areas enclosed in the dotted boxes.

To begin, the transverse movement of the atoms of β phase in the 14 Nb alloy was confirmed. Figure 6a,b shows the SADPs for the 14 Nb alloy.

Note that the pattern shown in Figure 6b is an approximately 7° tilt of that shown in Figure 6a. Interestingly, rod-like streaks and diffuse satellites at ±1/2[110]* can be observed in Figure 6a, but not in Figure 6b. The disappearance of the diffuse satellites in Figure 6b implies that double diffraction led to the appearance of these satellites. The disappearance of the diffuse satellites at ±1/2[110]* became more apparent after comparing the line intensity profiles of the dotted boxed regions, which are shown in Figure 6a′,b′. These findings indicate that the movement of the atoms of the β phase in the 14 Nb alloy was a transverse wave.

**Figure 7:**
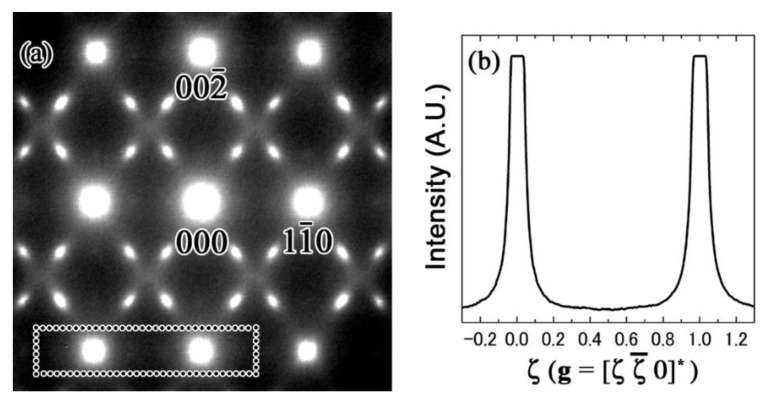
(**a**) SADP of the Zr-14 at.% Nb alloy (zone axis of [110]); (**b**) intensity profile along [110].

It should be noted that these satellites and rod-like streaks were not observed in the (110) SADP, as can be seen in Figure 7a. The disappearance of these satellites is evident in the intensity profile results shown in Figure 7b.

From these results, the orientation of the atom movement was determined. Particularly, considering that the movement of atoms was responsible for the occurrences of the diffuse satellites and rod-like streaks, we expected that the diffuse satellites and rod-like streaks would be missing in the special case. In this case, the inner product of the scattering vector of diffuse satellites, ***g***, and the displacement of atoms, ***R***, is zero. This means that ***g***·***R*** = 0. We can rewrite the scattering vector of diffuse satellite as ***g*** = ***g***_β_ + ***q***, where ***g***_β_ is the scattering vector of the β phase and ***q*** is the propagation vector for the displacement of atoms ***R***. In the case of the 14 Nb alloy, ***R*** is given by a transverse wave, as has been previously described. The inner product of ***q***·***R*** is always zero because this condition defined a transverse wave. Thus, the relation ***g***·***R*** = 0 is only satisfied when ***g***_β_·***R*** = 0. If ***R*** is the [001] direction, no diffuse reflections appear in the SADP when zone axis is [001], as shown in Table 2.

However, diffuse satellites and rod-like streaks appeared in the SADP when the zone axis is [001], as can be seen in Figure 6a. This implies that ***R*** was not [001] direction. When the displacement of atoms occurred in the [110] direction, the satellite reflections and streaks were not observed in the SADPs showing the [110] zone axis; however, they were present in the SADPs when the zone axis are [113] or [001], as can be seen in Figure 7. This is more apparent in the line intensity profiles corresponding to the boxed region in Figure 7a (i.e., in Figure 7b). Thus, the satellites at ***g***_β_ + <ζ$\bar{\zeta }$0>* were present because of the transverse wave consistent with the displacement of atoms in the <110> direction, which was perpendicular to the propagation vector ***q*** = <ζζ0>*, for ζ~1/2.

**Figure 8:**
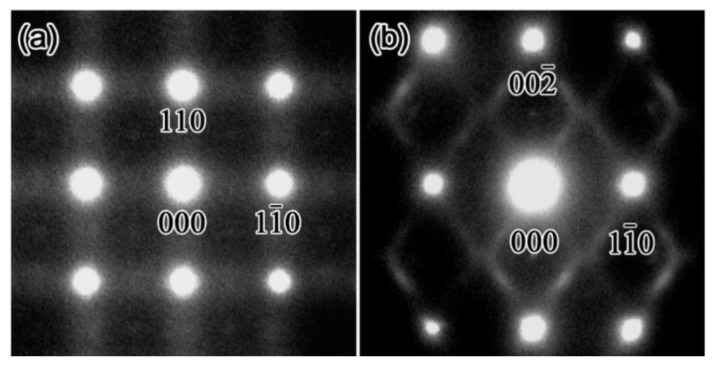
Diffraction patterns of Zr-18 at.%Nb alloy. The zone axis is [001] in (**a**) and [110] in (**b**).

Figure 8 shows the SADPs of the 18 Nb alloy at room temperature; in this case, a positive temperature dependence of the resistivity can be observed.

**Figure 9:**
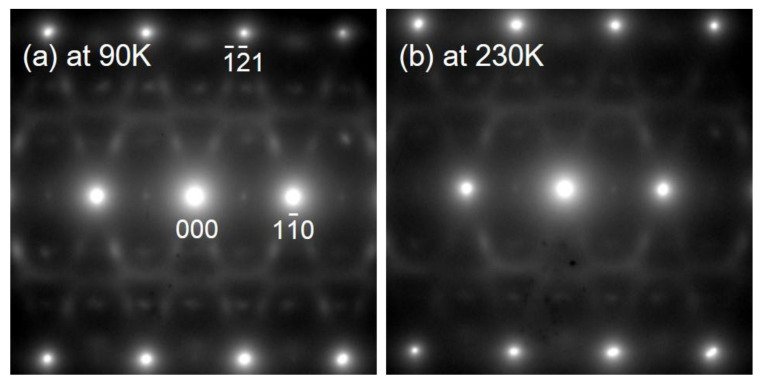
Temperature dependence of diffraction patterns of Zr-18 at.%Nb alloy: (**a**) taken at 90 K and (**b**) taken at 230 K.

The results shown in Figure 8 confirm weak lattice modulation in the 18 Nb alloy, as rod-like streaks in the <110>* direction can be observed in the (001) SADP, but not in the (110) SADP. However, no diffuse satellites were observed at ***g***_β_ + <ζζ0>* in the (001) SADP. It is possible that, in the case of Zr-Nb alloys, the diffuse satellites at ***g***_β_ + <ζζ0>* are also related to the NTC, which can be determined from the resistivity curve, and have a temperature dependence similar to that observed for Ti-Nb alloys [31]. As previously mentioned, the lattice modulation that occurs in Zr-Nb alloys is similar to the β phase in Ti alloys [26,31]. In fact, when the diffraction patterns in the 18 Nb alloy were investigated below *T*_min_ (= 240 K), the intensities of the ω phase reflections and diffuse satellites were stronger, as can be seen in Figure 9.

In Ti-Nb alloys, the β phase with lattice modulation presents as nanoscale-domain-like structures (i.e., nanodomains) [35]. It is possible that increasing the interface, such as the athermal ω phase and/or nanodomains, and the matrix β phase as the temperature is decreased coincidentally increases the electrical resistivity during the cooling process. Thus, the NTC is considered to have been attributable to the presence of an athermal ω phase and the lattice modulation in the β phase. It has previously been reported that the lattice modulation that occurs in the β phase in Ti alloys is related to the β phase stability, and is thus indicative of the softening of the elastic stiffness, *c’*, which contributes to reduce the Young’s modulus in the <001> direction [22,23,24,25,26]. In this study, transverse lattice modulation was found to occur in the β phase of the Zr-xNb alloys. The occurrence of this transverse wave may imply the softening of the elastic stiffness *c*’. It is necessary to minimize the Young’s modulus of metallic biomaterials to prevent bone degradation and absorption; this undesirable outcome is also known as stress shielding, and it occurs when there is a difference between the Young’s modulus of metallic implants and that of human bone [36,37,38].

By controlling the phase stability and matching the loading axis orientation with the <001> orientation, Zr-Nb-based metallic biomaterials with low Young’s moduli can be developed. Recently, additive manufacturing has enabled the simultaneous control of the shapes and textures of metallic materials [39,40,41,42,43,44]. Applying such processes in combination with the phase stability control of Zr-Nb alloys has led to the development of custom-made implants with low Young’s moduli.

**Figure 10:**
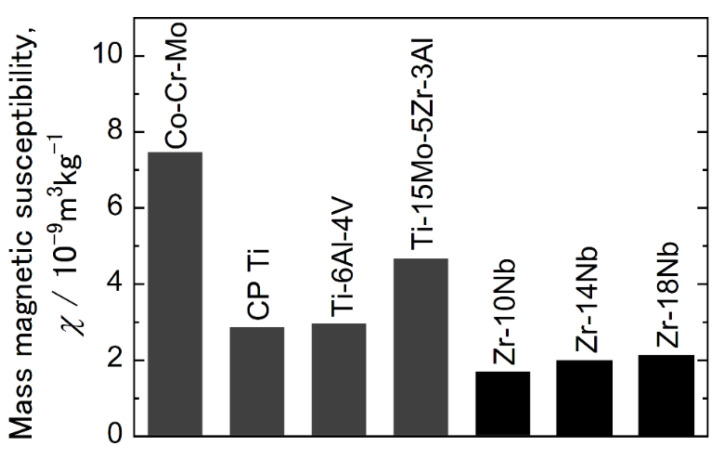
Magnetic susceptibilities of several alloys at room temperature [8].

Figure 10 shows the room-temperature magnetic susceptibilities of 10 Nb, 14 Nb and 18 Nb alloys. The magnetic susceptibilities of several other metallic biomaterials are also shown [8]. As can be seen, the magnetic susceptibility values for the Zr-Nb alloys were lower than those for the Co-Cr-Mo and Ti alloys.

**Figure 11:**
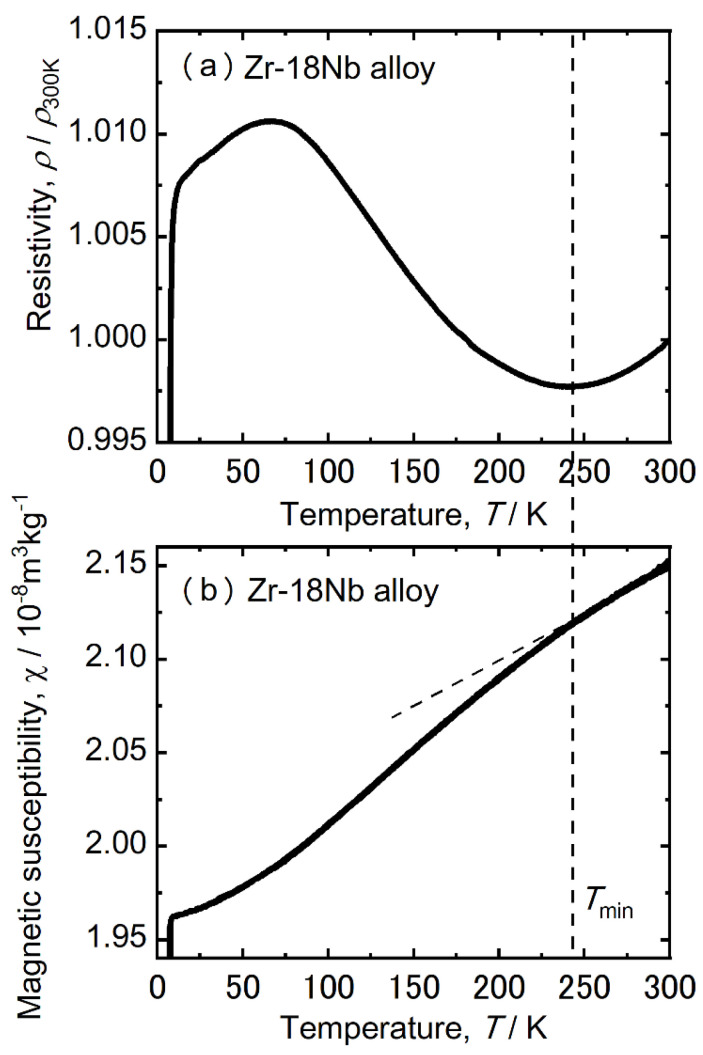
Electrical resistivity (**a**) and magnetic susceptibility (**b**) of the Zr-18 at.% Nb alloy.

The magnetic susceptibility values for the 10 Nb, 14 Nb, and 18 Nb alloys were found to be in good agreement with those reported by Nomura et al. [8]. Nomura et al. also reported that the magnetic susceptibility decreased when the thermal ω phase precipitated in the Zr-16 mass% Nb alloy. In this study, the lowest magnetic susceptibility was found in the 10 Nb alloys with the most ω phase precipitation. Thus, the low magnetic susceptibility of Zr alloys has been attributed to the high volume fraction of the ω phase. However, because the precipitation of the ω phase significantly embrittled the Zr-Nb alloy, its magnetic susceptibility and mechanical properties must be well balanced. Figure 11 shows the temperature dependence of electrical resistivity and magnetic susceptibility of the 18 Nb alloy.

It should be noted that the magnetic susceptibility decreased below *T*_min_. The temperature dependence of the athermal ω phase and lattice modulation likely contributed to the decrease in the magnetic susceptibility. Thus, Zr-Nb alloys with low magnetic susceptibility, superior mechanical properties, and low Young’s modulus can be realized by controlling the ω phase transformation and the lattice modulation. It was previously reported that as-cast alloys with 14 ≤ x ≤ 20 have superior ductility and a low Young’s modulus [9]. In this study, the occurrences of athermal ω phase transformation and lattice modulation in Zr-Nb alloys were found to be dependent on temperature and Nb content. This finding suggests that the volume fraction of the athermal ω phase and lattice modulation in Zr-Nb alloys can be controlled with relative ease. Thus, Zr-Nb alloys can be considered to be good MRI-compatible materials.

## 5. Conclusions

The relationship between the metastable phase and magnetic susceptibility of Zr-Nb alloys was investigated by conducting electrical resistivity measurements, magnetic susceptibility measurements, and TEM observations, focusing on an anomalous NTC in the resistivity curve, and the occurrence of the athermal ω phase and lattice modulation. The resistivity curves for the 10 Nb and 12 Nb alloy revealed a positive temperature coefficient at temperatures below 300 K. The resistivity curves for the 14 Nb and 18 Nb alloys revealed an anomalous NTC. Diffuse satellites were observed at ***g***_β_ + ***q*** in the SADPs of the 14 Nb alloy when zone axis was [113] and [001]; however, this was not the case when the zone axis was [110]. This result implies that the movement of atoms can be described as a transverse wave with a propagation vector of ***q*** = <ζζ0>*, for ζ~1/2 and displacement in the <110> direction. Furthermore, these diffuse satellites did not appear when the temperature coefficient was positive. Overall, the results indicate that low magnetic susceptibility and a relatively low Young’s modulus can be achieved by controlling the volume fraction of the athermal ω phase and the occurrence of lattice modulation. This means that Zr-Nb alloys can be manufactured to have low magnetic susceptibility, good deformability, and a low Young’s modulus by controlling the microstructure. Thus, Zr-Nb alloys are expected to be a next-generation MRI-compatible metallic biomaterial for medical devices.

## Figures and Tables

**Table 1:** Chemical composition of Zr-x at.% Nb (x = 10, 12, 14, and 18) alloy master ingots.

Nomial Composition	Chemical Composition (at.%)	
	Zr	Nb
Zr-10at.%Nb	Bal.	9.88
Zr-12at.%Nb	Bal.	12.12
Zr-14at.%Nb	Bal.	13.54
Zr-18at.%Nb	Bal.	17.88

**Table 2:** Appearance or disappearance of diffuse satellites for combinations of examined zone axis and [001], [110], and [111] displacement direction ***R***.

Direction of R	Zone Axis // [113]	Zone Axis // [001]	Zone Axis // [110]
[001]	Present	Absent	Present
[110]	Present	Present	Absent
[111]	Present	Present	Present

## Data Availability

Not applicable.

## References

[1] Olsen O.E. (2008). Practical body MRI—A paediatric perspective. Eur. J. Radiol..

[2] Olsrud J., Lätt J., Brockstedt S., Romner B., Björkman-Burtscher I.M. (2005). Magnetic resonance imaging artifacts caused by aneurysm clips and shunt valves: Dependence on field strength (1.5 and 3 T) and imaging parameters. J Magn. Reson. Imag..

[3] New P.F., Rosen B.R., Brady T.J., Buonanno F.S., Kistler J.P., Burt C.T., Hinshaw W.S., Newhouse J.H., Pohost G.M., Taveras J.M. (1983). Potential hazards and artifacts of ferromagnetic and nonferromagnetic surgical and dental materials and devices in nuclear magnetic resonance imaging. Radiology.

[4] Thomsen P., Larsson C., Ericson L.E., Sennerby L., Lausmaa J., Kasemo B. (1997). Structure of the interface between rabbit cortical bone and implants of gold, zirconium and titanium. J. Mater. Sci. Mater. Med..

[5] Eisenbarth E., Velten D., Müller M., Thull R., Breme J. (2004). Biocompatibility of beta-stabilizing elements of titanium alloys. Biomaterials.

[6] Yamamoto A., Honma R., Sumita M. (1998). Cytotoxicity evaluation of 43 metal salts using murine fibroblasts and osteoblastic cells. J. Biomed. Mater. Res..

[7] Zhou F.Y., Wang B.L., Qiu K.J., Lin W.J., Li L., Wang Y.B., Nie F.L., Zheng Y.F. (2012). Microstructure, corrosion behavior and cytotoxicity of Zr-Nb alloys for biomedical application. Mater. Sci. Eng. C.

[8] Nomura N., Tanaka Y., Kondo R., Doi H., Tsutsumi Y., Hanawa T. (2009). Effect of phase constitution of Zr-Nb alloys on their magnetic susceptibilities. Mater. Trans..

[9] Kondo R., Nomura N., Tsutsumi Y., Doi H., Hanawa T. (2011). Microstructure and mechanical properties of as-cast Zr-Nb alloys. Acta Biomater..

[10] Xia H., Parthasarathy G., Luo H., Vohra Y.K., Ruoff A.L. (1990). Crystal structures of group IVa metals at ultrahigh pressures. Phys. Rev. B Condens. Matter.

[11] Xia H., Duclos S.J., Ruoff A.L., Vohra Y.K. (1990). New high-pressure phase transition in zirconium metal. Phys. Rev. Lett..

[12] Sikka S.K., Vohra Y.K., Chidambaram R. (1982). Omega phase in materials. Prog. Mater. Sci..

[13] Silcock J.M. (1958). An X-ray examination of the ω phase in TiV, TiMo and TiCr alloys. Acta Metall..

[14] Hatt B.A., Roberts J.A. (1960). The ω-phase in zirconium base alloys. Acta Metall..

[15] De Fontaine D., Paton N.E., Williams J.C. (1971). The omega phase transformation in titanium alloys as an example of displacement controlled reactions. Acta Metall..

[16] Sass S.L. (1969). The ω phase in a Zr-25 at.% Ti alloy. Acta Mater..

[17] Benites G.M., Guillermet F., Cuello G.J., Campo J. (2000). Structural properties of metastable phases in Zr-Nb alloys: I. Neutron diffraction study and analysis of lattice parameter. J. Alloys Comp..

[18] Nomura N., Oya K., Tanaka Y., Kondo R., Doi H., Tsutsumi Y., Hanawa T. (2010). Microstructure and magnetic susceptibility of as-cast Zr-Mo alloys. Acta Biomater..

[19] Okunishi E., Kawai T., Mitsuhara M., Farjami S., Itakura M., Hara T., Nishida M. (2013). HAADF-STEM studies of athermal and isothermal ω-phase in β-Zr alloy. J. Alloy. Comp..

[20] Kondo R., Tsutsumi Y., Doi H., Nomura N., Hanawa T. (2011). Effect of phase constitution on magnetic susceptibility and mechanical properties of Zr-rich Zr-Mo alloys. Acta Biomater..

[21] Dey G.K., Tewari R., Jyoti G., Gupta S.C., Joshi K.D., Sikka S.K. (2004). Formation of a shock deformation induced ω phase in Zr 20 Nb alloy. Acta Mater..

[22] Lee S.H., Todai M., Tane M., Hagihara K., Nakajima H., Nakano T. (2012). Biocompatible low Young’s modulus achieved by strong crystallographic elastic anisotropy in Ti-15Mo-5Zr-3Al alloy single crystal. J. Mech. Behav. Biomed. Mater..

[23] Tane M., Akita S., Nakano T., Hagihara K., Umakoshi Y., Niinomi M., Nakajima H. (2008). Peculiar elastic behavior of Ti-Nb-Ta-Zr single crystals. Acta Mater..

[24] Tane M., Akita S., Nakano T., Hagihara K., Umakoshi Y., Niinomi M., Mori H., Nakajima H. (2010). Low Young’s modulus of Ti-Nb-Ta-Zr alloys caused by softening in shear modului c′ and c44 near lower limit of body-centered cubic phase stability. Acta Mater..

[25] Wang P., Todai M., Nakano T. (2019). Beta titanium single crystal with bone-like elastic modulus and large crystallographic elastic anisotropy. J. Alloy. Comp..

[26] Wang P., Todai M., Nakano T. (2018). ω-phase transformation and lattice modulation in biomedical β-phase Ti-Nb-Al alloys. J. Alloy. Comp..

[27] Wang P., Todai M., Nakano T. (2013). β phase instability in Binary Ti-xNb Biomaterial single crystal. Mater. Trans..

[28] Ames S.L., McQuillan A.D. (1954). The resistivity-temperature-concentration relationships in the system niobium-titanium. Acta Metall..

[29] Ikeda M., Komatsu S., Sugimoto T., Kamei K. (1988). Temperature range of formation of athermal ω phase in quenched β Ti-Nb alloys. J. Jap. Ins. Metals..

[30] Ho J.C., Collings E.W. (1972). Anomalous electrical resistivity in titanium-molybdenum alloys. Phys. Rev. B.

[31] Todai M., Fukuda T., Kakeshita T. (2013). Relation between negative temperature coefficient in electrical resistivity and athermal ω phase Ti-xNb (26 ≤ x ≤ 29 at.%) alloys. J. Alloys Compd..

[32] Kuan T.S., Sass S.L. (1977). The direct imaging of a linear defect using diffuse scattering in Zr-Nb b.c.c. solid solutions. Philos. Mag..

[33] Todai M., Fukuda T., Kakeshita T. (2014). Temperature dependence of diffuse satellites in Ti–(50−x)Pd–xFe (14 ≤ x ≤ 20 (at.%)) alloys. J. Alloys Compd..

[34] Todai M., Fukuda T., Kakeshita T. (2013). Direction of atom displacement in incommensurate state of Ti–32Pd–18Fe shape memory alloy. Mater. Lett..

[35] Tahara M., Kim H.Y., Inamura T., Hosoda H., Miyazaki S. (2011). Lattice modulation and superelasticity in oxygen-added β-Ti alloys. Acta Mater..

[36] Noyama Y., Miura T., Ishimoto T., Itaya T., Niinomi M., Nakano T. (2012). Bone Loss and Reduced Bone Quality of the Human Femur after Total Hip Arthroplasty under Stress-Shielding Effects by Titanium-Based Implant. Mater. Trans..

[37] Nakano T., Kaibara K., Ishimoto T., Tabata Y., Umakoshi Y. (2012). Biological apatite (BAp) crystallographic orientation and texture as a new index for assessing the microstructure and function of bone regenerated by tissue engineering. Bone.

[38] Matsugaki A., Aramoto G., Nakano T. (2012). The alignment of MC3T3-E1 osteoblasts on steps of slip traces introduced by dislocation motion. Biomaterials.

[39] Ishimoto T., Ozasa R., Nakano K., Weinmann M., Schnitter C., Stenzel M., Matsugaki A., Nagase T., Matsuzaka T., Todai M. (2021). Development of TiNbTaZrMo bio-high entropy alloy (BioHEA) super-solid solution by selective laser melting, and its improved mechanical property and biocompatibility. Scr. Mater..

[40] Ishimoto T., Hagihara K., Hisamoto K., Sun S.H., Naknao T. (2021). Crystallographic texture control of beta-type Ti–15Mo–5Zr–3Al alloy by selective laser melting for the development of novel implants with a biocompatible low Young’s modulus. Scr. Mater..

[41] Gokcekaya O., Hayashi N., Ishimoto T., Ueda K., Narushima T., Nakano T. (2020). Crystallographic orientation control of pure chromium via laser powder bed fusion and improved high temperature oxidation resistance. Addit. Manuf..

[42] Nagase T., Hori T., Todai M., Sun S.H., Nakano T. (2019). Additive manufacturing of dense components in beta-titanium alloys with crystallographic texture from a mixture of pure metallic element powders. Mater. Des..

[43] Todai M., Nakano T., Liu T., Yasuda H.Y., Hagihara K., Cho K., Ueda M., Takeyama M. (2017). Effect of building direction on the microstructure and tensile properties of Ti-48Al-2Cr-2Nb alloy additively manufactured by electron beam melting. Addit. Manuf..

[44] Nomura N., Kawasaki A. (2021). Development of low magnetic zirconium-based alloys and the additive manufactured builds for biomedical applications. J. Jpn. Soc. Pow. Pow. Metall..

